# Adalimumab biosimilars, ABP501 and SB5, are equally effective and safe as adalimumab originator

**DOI:** 10.1038/s41598-021-89790-4

**Published:** 2021-05-14

**Authors:** Linda Cingolani, Brigida Barberio, Fabiana Zingone, Antonio Ferronato, Lorenzo Bertani, Francesco Costa, Giorgia Bodini, Maria Giulia Demarzo, Piera Melatti, Alessandro Gubbiotti, Davide Massimi, Cesare Casadei, Renata D’Incà, Edoardo Vincenzo Savarino

**Affiliations:** 1 Division of Gastroenterology, Department of Surgery, Oncology and Gastroenterology (DiSCOG), University of Padua, Via Giustiniani 2, 35121 Padua, Italy; 2 Endoscopy Unit, Alto Vicentino Hospital, AULSS7, Pedemontana, Italy; 3 Department of Translational Research and New Technologies in Medicine and Surgery, University of Pisa, Pisa, Italy; 4 Gastroenterology Unit, Department of Internal Medicine, IRCCS Ospedale Policlinico San Martino, University of Genoa, Genoa, Italy

**Keywords:** Drug discovery, Immunology, Medical research

## Abstract

To date, data on effectiveness and safety of Adalimumab (ADA) biosimilars in inflammatory bowel diseases (IBDs) are lacking. Therefore, we aimed to verify the ability of ABP501 and SB5 to maintain the clinical and biochemical response induced by the ADA originator, after switching to them. We prospectively analyzed data collected from 55 patients with IBD who switched to ABP501, and 25 patients with IBD who switched to SB5, from ADA originator at four IBD Units between 2018 and 2020. In addition, we included an age and sex-matched control group (n = 38) who continued ADA originator for at least two years and who did not switch to a biosimilar drug. Clinical and biochemical data (C-Reactive Protein (CRP), fecal calprotectin (FC)), concomitant steroid and/or immunosuppressant therapy at the time of the switch and after six months were collected. At six months, in the ABP501 group, we did not observe statistically significant modifications in clinical activity of disease (p = 0.09) and FC values (p = 0.90)_._ Some patients (n = 8) needed to add steroids at six months after switching (p = 0.01), however the need for optimization was not significant between the two timepoints (p = 0.70). Finally, 14.5% patients stopped therapy after six months. Similarly, in the SB5 group we observed a stability of clinical activity and FC values (p = 0.90 and p = 0.20), and a concomitant statistically significant decrease in CRP (p = 0.03). There were no differences in steroids/immunosuppressants need or optimizing biological therapy in this group. Finally, drug survival curves of patients who switched from originator to ABP501 and those who continued ADA originator were similar (p = 0.20). Overall, biosimilar drugs seem to be as effective and safe as the originator. Further larger and longer studies are mandatory to understand the clinical implications of these findings.

## Introduction

Inflammatory Bowel Diseases (IBDs), including ulcerative colitis (UC) and Crohn’s Disease (CD), are chronic inflammatory disorders which are characterized by intermittent periods of remission and relapses^1,2^, and can greatly affect quality of life (QoL) of patients^3–5^. Prognosis dramatically changed after the introduction of biological agents, including anti-Tumor Necrosis Factor-alpha (TNF-α)^6^, which can control flares, prevent complications and halt disease progression^7,8^. Among these agents, Adalimumab (ADA) originator, Humira, a fully human IgG1 monoclonal antibody directed against TNF-α, was introduced in 2002 and soon became one of the mainstays of therapy for moderate to severe IBD^7,9–11^.

Since expiration of its patent in 2016, several biosimilars have been developed for clinical use. A biosimilar is a biologic drug that is highly similar, in terms of clinical behavior (including efficacy, safety, immunogenicity, and pharmacokinetics) and product quality, to a previously approved existing biologic therapy known as the reference product (RP)^12^. ABP501 (Amgevita) was the first approved biosimilar to ADA by the Food and Drug Administration (FDA) in 2016 and by the European Medicines Agency (EMA) in 2017 for all the requested indications of ADA^13^. One year later, in 2017, SB5 (Imraldi) biosimilar was approved by the EMA as well^14^.

The regulatory approval of ADA biosimilars for the treatment of IBD patients was based on data derived from trials on rheumatologic patients^15^. In fact, EMA exploited the principle of extrapolation: this is the extension of efficacy and safety data originated from an already approved therapeutic indication for which the biosimilar has been clinically tested, to other indications for which the innovator product has been previously authorized^16^. That is one of the reasons why, as with other anti-TNF biosimilars already on the market^17^, real-life data and pharmacovigilance studies are needed to develop long-term evidence on efficacy and safety in IBD population.

To date, current literature is still lacking in studies evaluating efficacy and safety of biosimilar therapies in patients with IBD, while some more data are available in rheumatological and dermatological field^18–20^. Therefore, we aimed to prospectively verify as primary outcome the ability of biosimilar ABP501 and biosimilar SB5 to maintain clinical and biochemical response induced by ADA originator after switching to them in an Italian multicentric cohort of IBD patients. As secondary outcome we aimed to verify the data about safety and tolerability of both ADA biosimilars compared to their ADA originator.

## Methods

This is a multicenter prospective cohort study, coordinated by the IBD Unit of Padua University, with the involvement of other three Italian IBD centers (Santorso, Pisa, and Genoa). Overall, IBD patients in therapy with ADA originator (Humira), were prospectively enrolled and switched to the biosimilars ABP501 (Padua, Santorso) or SB5 (Genoa, Pisa) between October 2018 and July 2020 after an educational visit (Switch Groups). Thereafter, we matched them by age and sex with consecutive patients who continued ADA originator for at least two years (i.e. median time with ADA originator of the switch population before changing to biosimilars) and who did not switch to a biosimilar (Non-Switch Group). The study was approved by University of Padova’s Ethics Committee as part of a larger study aimed to evaluate disease course and characteristics of IBD patients from the introduction of biologics in clinical practice (N. 3312/AO/14). Written informed consent was obtained from all eligible participants or their legal representatives before participation. The study protocol was performed accordingly to the ethical guidelines of the 1964 Declaration of Helsinki (6th revision, 2008) as reflected in a priori approval by the institution’s human research committee.

To note, we analyzed separately the switch to the two biosimilars. We evaluated clinical and biochemical features at baseline (before switching, T_0_), and after 6 months (T_1_). The following baseline data were collected for each patient: age, sex, date of start of the ADA originator, dose of the last ADA originator administration, value of fecal calprotectin, use of steroids, use of immunosuppressants (azathioprine, 6-mercaptopurine, methotrexate) and adverse events. Clinical activity was measured by using partial Mayo (p-Mayo) Score and Harvey-Bradshaw Index (HBI) for UC and CD, respectively. According to medical literature, clinical activity was classified into remission, mild, moderate and severe according to the following values of pMayo for UC: 0–1 remission, 2–4 mild disease, 5–6 moderate disease, 7–9 severe. Conversely, the following values of HBI were used for CD patients: < 5 remission, 5–7 mild disease, 5–15 moderate, > 16 severe. C-Reactive Protein (CRP) levels as dichotomous feature (positive or negative) and fecal calprotectin (FC) were also evaluated at the same timepoints^21^. Optimization rate for both the ADA originator and biosimilar was recorded at T_0_ and T_1_. The allowed methods of therapeutic optimization were: 40 mg every week or 80 mg every 2 weeks.

Finally, *treatment failure* was defined as discontinuation of biological therapy due to adverse events (AEs), lack of clinical response and need for hospitalization/surgery. AEs which lead to discontinuation of therapy, were also recorded.

### Statistical analysis

The data were analyzed using the STATA11 software (Stata Corp., College Station, TX, USA)^22^. Continuous variables were reported as medians with range of values, categorical variables were reported as frequencies and percentages. To determine if there is a statistically significant difference in proportion between paired data we used *McNemar’s Test,* while the comparison between ordinal or continuous values over the study period (T0 vs T1) was performed using the Wilcoxon signed-rank test. Comparison between Switch Group (ABP501) and Non-Switch Group was carried out using Mann–Whitney test for numerical data and χ^2^ test for categorical data. The statistical significance was set for values of p ≤ 0.05.

### Ethics committee approval

The study was approved by University of Padova’s Ethics Committee as part of a larger study aimed to evaluate disease course and characteristics of IBD patients from the introduction of biologics in clinical practice (N. 3312/AO/14).

### Informed consent

Informed consent was obtained from all individual participants included in the study.

## Results

We enrolled 55 patients with IBD who switched therapy from ADA originator to biosimilar drug ABP501 (T_0_) and completed at least six months of therapy with ABP501 (T_1_). The main characteristics of this population were provided in Table 1. A smaller group of 25 patients who switched from ADA originator to SB5 with the same demographic and clinical characteristics was also analyzed. For comparison, 38 sex- and age-matched patients who continued ADA originator for at least two years and who did not switch to a biosimilar were included (Table 1).

**Table 1:** Main characteristics of the control group (non-switch group) at baseline (T0) and of population switched to ABP501 biosimilar (switch group) at baseline (before switching, T0).

	Switch group (ABP501) at baseline (n = 55)	Non-switch group (originator) at baseline (n = 38)	P value
Male gender, n (%)	35 (63.6)	28 (73.7)	0.38
Median age at baseline, years (25th–75th perc)	47.4 (43.4–51.3)	47.4 (43.2–51.5)	0.97
Median time from adalimumab start, years (25th–75th perc)	3.4 (2.1–4.7)	3.5 (2–4.5)	0.91
**Type of disease, n (%)**			
CD	45 (81.8)	25 (65.8)	0.08
UC	10 (18.2)	13 (34.2)	
**Phenotypes of Crohn’s disease patients, n (%)**			
Penetrating	4 (8.9)	(4)	
Stenotic	18 (40)	6 (24)	0.35
Stenotic and penetrating	4 (8.9)	2 (8)	
Inflammatory	19 (42.2)	16 (64)	
**Localization of Crohn’s disease, n (%)**			
Colon	4 (8.9)	4 (16)	
Ileum-colon	30 (66.7)	68)	
Ileum	9 (20)	2 (8)	0.46
Upper GI	–	–	
Upper + other	2 (4.4)	2 (8)	
**Localization of ulcerative colitis, n (%)**			
Proctitis	2 (20)	0	0.20
Left colon	3 (30)	6 (46.1)	
Pancolitis	5 (50)	7 (53.8)	

### Clinical and biochemical data after 6 months from the switch to ABP501 biosimilar

**Figure 1:**
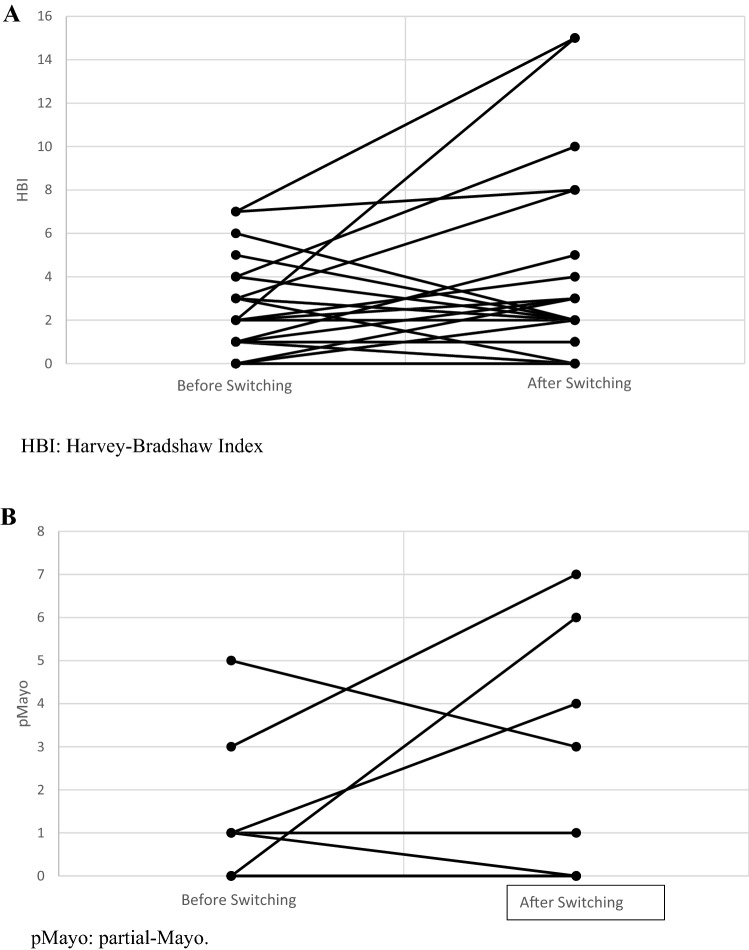
Clinical disease activity in patients affected by Crohn’s disease (**A**) and ulcerative colitis (**B**) at baseline and six months after switching from originator to ABP501.

The majority of patients were affected by CD (N = 45, 81.8%) and were men (N = 35, 63.6%). At baseline, based on clinical scores: 47 (85.5%) patients were in remission; 5 (9.1%) had a mild disease; 3 (5.4%) had a moderate activity of disease; none had severe disease. We reassessed the disease clinical activity after 6 months from switching therapy: we found that 42 patients (76.4%) were still in remission; 4 (7.3%) had a mild disease; 8 (14.5%) had a moderate disease and one (1.8%) had a severe disease (T_0_ vs T_1_: p = 0.09) (Table 2; Fig. 1A, B).

**Table 2:** Clinical and biochemical activity, drug dosages and reason for stopping drug of population switched to ABP501 biosimilar (Switch Group) at baseline (before switching, T0) and at six months after switching (T1).

	Switch group (ABP501)		
	Baseline (T0)	At 6 months (T1)	P value T0 vs T1
Disease clinical activity, n (%)*			
Remission	47 (85.4)	42 (76.3)	0.09
Mild	5 (9.1)	(7.3)	
Moderate	3 (5.4)	8 (14.5)	
Severe	–	1 (1.8)	
Fecal calprotectin, µg/g (median, 25th–75th percentile)	53 (17–139)	50 (19–135)	0.90
Positive C-reactive protein, n (%)	8 (14.5)	8 (14.5)	1.0
**Concomitant drugs, n (%)**			
Steroids	1 (1.8)	8 (14.5)	0.01
Azathioprine	10 (18.2)	10 (18.2)	1.00
**Adalimumab dosage administered, n (%)**			
40 mg/2 weeks	41 (74.5)	36 (65.5)	0.70
40 mg/1 week	14 (25.5)	(18.2)	
80 mg/2 weeks	–	1 (1.8)	
Stop adalimumab, n (%)	–	8 (14.5)	–
**Stop because of:**			
Lack of efficacy	–	6 (75.0)	–
Adverse reaction		1 (12.5)	
Need to surgery		1 (12.5)	

*According to HBI (Crohn’s disease) or pMayo score (ulcerative colitis).

At baseline median FC value was 53 µg/g (range 17–139 µg/g), while it was 50 µg/g (range 19–135) at six months, without statistically significant difference between T_0_ and T_1_ (p = 0.90). CRP values were still found positive in the same 8 (14.5%) patients who had abnormal values at baseline (p = 1.0). From baseline to 6 months, a statistically significantly (p = 0.01) increase of patients who needed to add steroid therapy (n = 8) to biological therapy was registered. On the contrary, immunomodulatory therapy with azathioprine was not significantly modified (p = 1.0) (Table 2).

**Figure 2:**
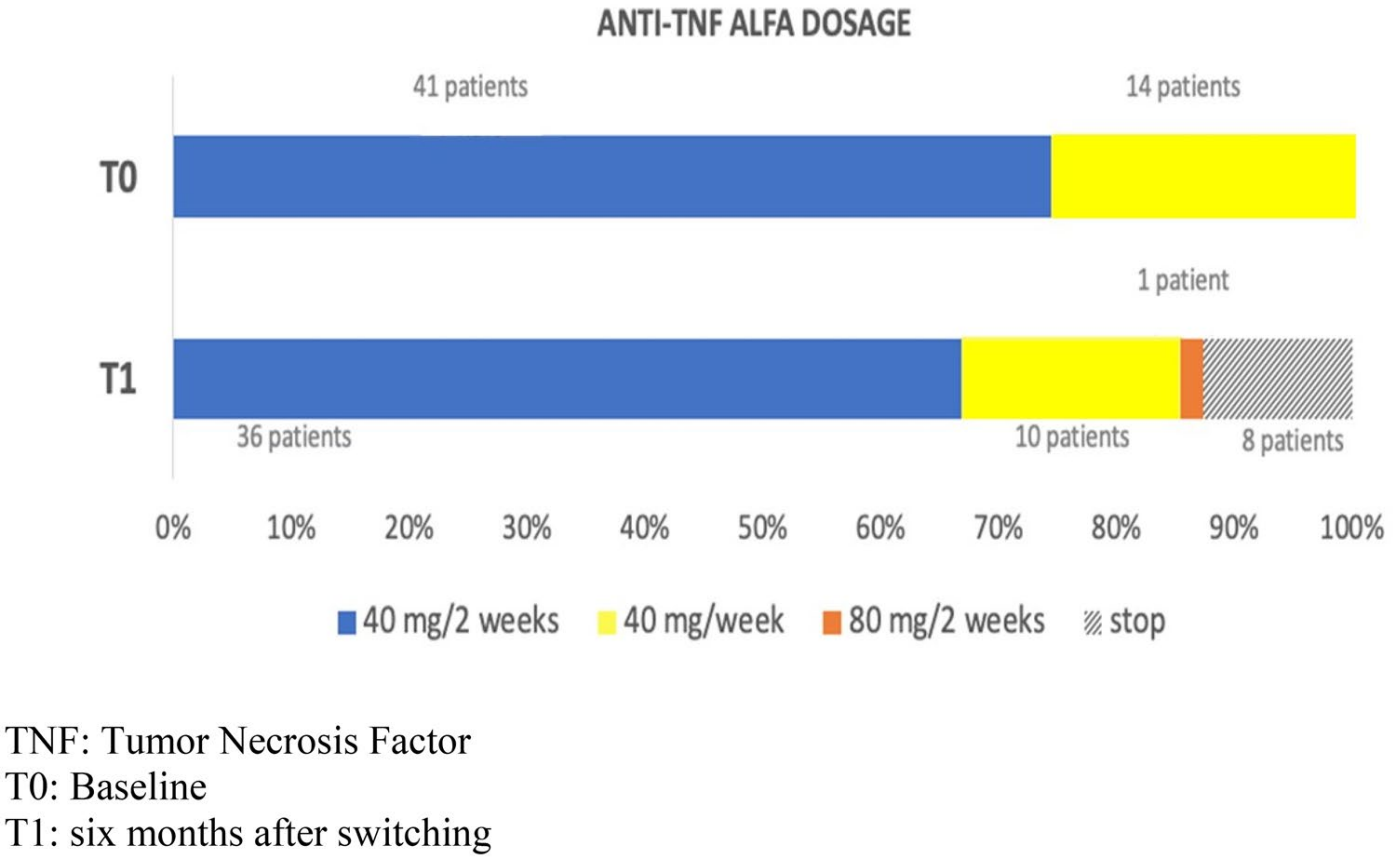
Anti-TNFα Dosage at baseline (T0) and six months after switching from originator to ABP501 (T1).

Regarding optimization rate of drug: 41 patients (74.5%) at T_0_ and 36 (65.5%) patients at T_1_ were taking 40 mg every 2 weeks; 14 (25.4%) patients at T_0_ and 10 (18.2%) patients at T_1_ were taking 40 mg every 2 weeks; and one (1.8%) patient at T_1_ was taking 80 mg every 2 weeks (Table 2; Fig. 2).

Overall, the need for optimization did not significantly increase (p = 0.70) from T_0_ to T_1._ After 6 months from switching, eight (14.5%) patients discontinued therapy: six for ineffectiveness, one for adverse reaction and one because of need for surgical operation.

**Figure 3:**
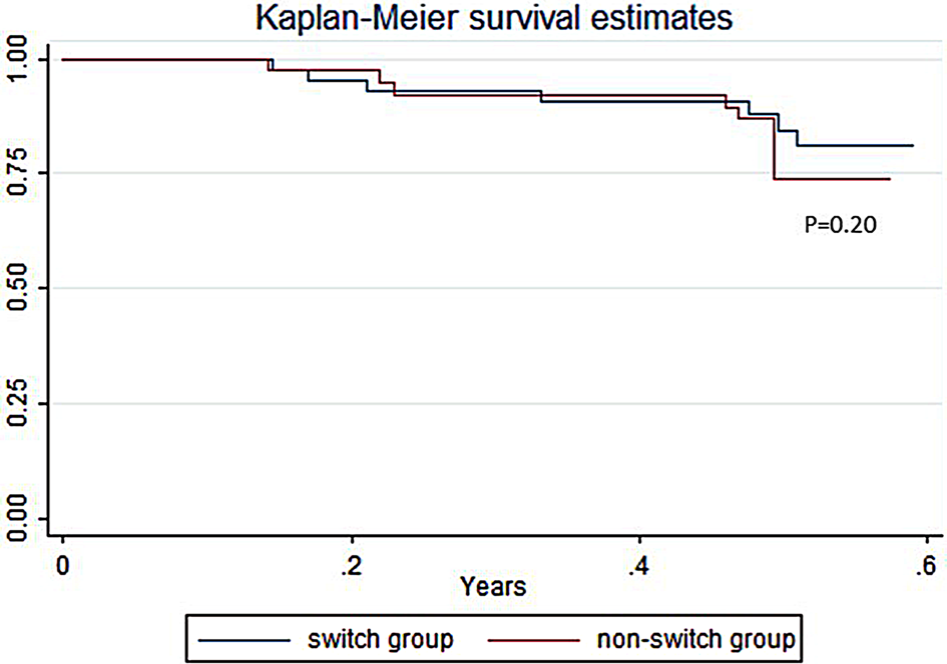
Kaplan–Meier drug survival curves of patients who switched from originator to ABP501 and those who continued ADA originator.

Finally, the Kaplan–Meier drug survival curves showed no statistically significant difference between patients who switched from originator to ABP501 and those who continued ADA originator (p = 0.20) (Fig. 3).

### Clinical and biochemical data after 6 months from the switch to SB5

The majority of patients were affected by CD (18; 72%) and 15 (60%) were men.

At baseline, based on clinical scores: 24 (96%) patients were in remission; one patient (4%) had a mild disease; none had moderate or severe clinical disease. At 6 months, we found that 21 (84%) patients maintained clinical remission, 3 (12%) had a mild disease, and one patient (4%) had a moderate disease, none had a severe disease. The difference between T_0_ and T_1_ was not statistically significant (p = 0.20).

Comparing baseline with 6 months, the median FC value at the time of the switch was 97 ug/g (range 42–276 ug/g) and 50 ug/g (range 0–166 ug/g) after 6 months (p = 0.20). Positive CRP values were found in 12 (48%) patients at baseline, with a statistically significant decrease after 6 months (n = 5, p = 0.03). We did not register a statistically significant difference in adding steroid or immunosuppressant therapy (respectively, p = 0.50 and p = 0.30). Regarding optimization rate of drug at baseline: 23 patients were in 40 mg every 2 weeks regimen (92%) and only 2 patients (8%) in 40 mg every week regimen. After 6 months we observed the same rates of optimized regimen, while one of the patients who were in 40 mg every 2 weeks regimen need to discontinue therapy because of an adverse event (T_0_ vs T_1_ p = 1.0).

## Discussion

The recent authorization of various ADA biosimilars for the treatment of IBD largely changed our daily clinical practice, creating new possibilities, but also some concerns about their use. Indeed, while we have significantly reduced the healthcare costs related to IBD treatments thanks to the adoption of these molecules, it is relevant to acknowledge that this was done without a consistent literature supporting their efficacy and safety in the gastroenterological field. Most of the data have been extrapolated from studies in rheumatology. Therefore, in our study we aimed to evaluate the effectiveness and tolerability of ADA biosimilar ABP501 and biosimilar SB5 in patients with IBD in a real-life setting, through the maintaining of clinical remission, dosage of therapy and biochemical features in the short-term (6 months) after switching from ADA originator.

Overall, we found that patients who switched from ADA originator to ABP501 and SB5 maintained the same clinical activity after 6 months from switching, without a significant need for drug optimization. Drug survival curves of patients who switched from originator to ABP501 biosimilar and of those who continued ADA originator showed no statistically significant difference between the two groups (p = 0.20), suggesting that switching does not affect drug administration and, indirectly, both effectiveness and safety. However, a significant percentage (n = 8, 14.5%; p = 0.01) of patients in the ABP501 population needed to temporarily add oral steroids to therapy, while, on the contrary, this finding was not statistically observed in SB5 population. Regarding biochemical scores, we did not find statistically significant differences of FC values between baseline and 6 months for both ADA biosimilars while we observed a significant decrease of CRP values in the SB5 group. Moreover, rates of optimization at 6 months were not significant both in the ABP501 and SB5 groups (respectively, p = 0.70 and p = 1.0) and in some patients we were even able to decrease the initial dosage, as a consequence of a stable clinical and biochemical disease remission. Regarding rates of therapy discontinuation, eight patients who switched to ABP501 needed to stop therapy (14.5%) mostly because of inefficacy (n = 6), one for adverse reaction and one because of need for surgery. Among patients switched to SB5, instead, we observed only one case of discontinuation for AE.

Looking at the existing literature, most of data regarding safety and efficacy of ADA biosimilars derived from studies conducted on rheumatological patients. In particular, recently, a systematic literature review^18^, including two large phase III randomized studies which sustained efficacy and tolerability of ABP501 and SB5 in Rheumatoid Arthritis^19,20^, confirmed the non-inferiority of ADA biosimilar drugs compared to the reference product. Currently, in fact, only few studies, with small populations included, directly evaluated the switching from ADA originator to ABP501 or SB5^23^. Ribaldone et al. evaluated the effectiveness and safety of ABP501 in 25 Italian patients with CD naïve to ADA, as well as the ABP501 maintenance in 62 patients switched from the ADA reference product^23^. In the latter group, drug retention after 6 months was observed in 59 out of 62 patients (95.2%), while the reason for discontinuation was secondary failure in all patients. The mean values of HBI at baseline did not change significantly after six months of therapy (p = 0.23), as well as the mean values of the CRP from baseline to six months of treatment (p = 0.32). In addition, they reported that the ABP501 dose was escalated in three patients (4.8%)^23^. In keeping with this study, our results did not show a statistically significant difference in clinical disease activity or in biochemical data from baseline to 6 months. However, in our study population a greater percentage of patients discontinued therapy (n = 8, 14.5%), mostly because of inefficacy, and a significant percentage of patients (n = 8, p = 0.01) needed to temporarily add steroids to therapy due to a flair of the disease as well.

Regarding SB5 biosimilar, a recent retrospective analysis from a single tertiary clinical center compared the outcomes of 93 patients with IBD who switched to SB5 with a control cohort of subjects treated with the ADA originator. Disease activity scores were not significantly different between the two groups at week 10 (p = 0.18 and p = 0.67, respectively). In addition, no significant differences of CRP and FC from baseline to week 10 between the two cohorts were observed. Likewise, in our patients treated with SB5 biosimilar, we found that clinical disease activity was comparable before and after six months from switching (p = 0.20), with a concomitant statistically significant decrease in CRP (p = 0.03). Another recent Italian study followed for 12 months patients with IBD who started SB5 treatment (naïve cohort) and those who underwent a switch from ADA originator to SB5 (switching cohort)^24^. In the naïve cohort, the overall remission rate at 12 months was 60.4%, whereas in the switching cohort it was 89.0%. In addition, they did not find differences in terms of ADA serum trough levels at baseline, 3 and 6 months after switching.

A strength of our study is that data were collected from multiple Gastroenterology Units, making the study population representative of multiple geographical areas and limiting single center clinical bias. Moreover, we included a sex- and age-matched group of control who continued ADA originator as further point of strength. The study has also some limitations to acknowledge. First of all, the modest sample size, with the risk of not having an adequate sensitivity to identify any causal effect, and moreover it may have led to over- or under- estimate some phenomena, such as the discontinuation therapy. Furthermore, the follow-up is not long enough to gather reliable data on the long-term response. Even more, the baseline population was heterogeneous, including biologic monotherapy and concomitant immunosuppressant and steroid therapy. This, however, reflects real life setting more than the cohorts selected for the registration trials. Finally, we did not assess serum trough levels and antidrug antibodies at baseline and during follow-up.

In conclusion, we found that ADA biosimilar drugs seem to be as effective as the originator, although a need for implementation of therapy with oral steroids was observed. This finding may imply a necessity of increased monitoring after switching therapy, in order to manage or prevent an exacerbation of the disease. Surely, biosimilars represent a great opportunity to reduce the costs of biological therapies and lighten the impact on healthcare resources^15,25^, but larger and longer studies in real-life are needed to improve our knowledge about their efficacy and safety in patients with IBD.

## Data Availability

The datasets used and/or analyzed during the current study are available from the corresponding author on reasonable request.

## Abbreviations

AEs: Adverse events
CD: Crohn’s disease
CRP: C-reactive protein
FC: Fecal calprotectin
HBI: Harvey–Bradshaw index
IBD: Inflammatory bowel disease
ADA: Adalimumab
p-MAYO: Partial Mayo score
UC: Ulcerative colitis
